# Genotype–Environment Interaction and Horizontal and Vertical Distributions of Heartwood for *Acacia melanoxylon* R.Br

**DOI:** 10.3390/genes14061299

**Published:** 2023-06-20

**Authors:** Ruping Zhang, Bingshan Zeng, Tianxiao Chen, Bing Hu

**Affiliations:** Research Institute of Tropical Forestry, Chinese Academy of Forestry, Guangzhou 510520, China; zrpzrpzrp@126.com (R.Z.); b.s.zeng@vip.tom.com (B.Z.); chentianxiao21@126.com (T.C.)

**Keywords:** *Acacia melanoxylon*, genotype–environment interactions, clonal repeatability, clones, sites, heartwood, sapwood, vertical variation, horizontal variation

## Abstract

*Acacia melanoxylon* (blackwood) is a valuable wood with excellent-quality heartwood extensively utilized worldwide. The main aim of this study was to confirm the horizontal and vertical variation and provide estimated values of genetic gains and clonal repeatabilities for improving the breeding program of *A. melanoxylon*. Six blackwood clones at 10 years old were analyzed in Heyuan and Baise cities in China. Stem trunk analysis was conducted for sample trees to explore the differences between heartwood and sapwood. The heartwood radius (HR), heartwood area (HA), and heartwood volume (HV) in heartwood properties decreased as the tree height (H) in growth traits increased, and the HV = 1.2502 DBH (diameter at breast height)^1.7009^ model can accurately estimate the heartwood volume. Furthermore, G × E analysis showed that the heritabilities of the eleven indices, including DBH, DGH (diameter at ground height), H, HR, SW (sapwood width), BT (bark thickness), HA, SA (sapwood area), HV, HRP (heartwood radius percentage), HAP (heartwood area percentage), and HVP (heartwood volume percentage) were between 0.94 and 0.99, and repeatabilities of the eleven indices were between 0.74 and 0.90. Clonal repeatability of DBH (0.88), DGH (0.88), and H (0.90) in growth traits and HR (0.90), HVP (0.90), and HV (0.88) in heartwood properties were slightly higher than for SA (0.74), SW (0.75), HAP (0.75), HRP (0.75), and HVP (0.75). These data also implied that the growth characteristics of heartwood and sapwood of blackwood clones were less affected by the environment and had substantial heritability.

## 1. Introduction

*Acacia melanoxylon*, known as blackwood, is one of the most important species in the Mimosaceae Acacia genus owing to its high-quality timber [1]. *A. melanoxylon* has a natural distribution spanning a vast area of approximately 3000 km from the southern to the northern regions of Australia. Currently, it is cultivated globally in Europe, Asia, South America, and Africa [2,3,4,5]. It was introduced to China in the early 1950s [2]. As part of the national reserve forest project to solve the existing problems of forest quality improvement and ecological environment restoration, it has been widely planted in South China.

Heartwood’s quantity and quality are the primary factors determining timber’s commercial value and quality [3]. When used in harsh environments, blackwood’s wood properties are well known for their decay resistance and durability [4]. The *A. melanoxylon* wood shows distinct growth rings with dense latewood bands. Occasionally, figures such as bird’s eye or fiddleback are exhibited in the heartwood. The plentiful heartwood extract also dramatically influences the color of heartwood [5]. For these qualities, heartwood has an extensive commercial application and shows strong demand in the international market, especially in manufacturing high-quality furniture, flooring, and musical instruments [6]. 

There are some references to the horizontal and vertical distributions of heartwood. Studies have also shown that the growth efficiency of heartwood and sapwood in the horizontal and vertical directions was different [7,8]. Yang predicted the heartwood radius, heartwood, and sapwood areas of *Tectona grandis* which showed significant differences in horizontal and vertical directions among trees [9]. Research on 35-year-old black walnut offspring illustrated that trees with faster growth rates possess more heartwood and a greater proportion of heartwood area (HVP) in a cross-section [10]. Therefore, knowing the differences between sapwood and heartwood attributes is meaningful. Until now, only Yang has illustrated the horizontal and vertical distributions of heartwood for the *T. grandis*, but for the clones, it is still unclear [9].

The phenotypic variation in plants results from their genotypes adapting to environmental changes. This variation is a permanent change that occurs over time due to prolonged selection pressure [11,12]. Research has demonstrated that *A. melanoxylon* is characterized by considerable fluctuations in basic density, wood properties, and heartwood color [3,5]. Despite much research on forest tree gene–environment interaction (G × E), the scope of clones examined remains relatively restricted [3,13,14]. Clones are expected to exhibit heightened sensitivity in the evaluation of genotypic stability and the detection of G × E interactions compared to family-origin plants, owing to the lack of genetic effects among ortet ramets and the significant non-additive genetic effects among clones [13,15]. According to earlier studies, the proportion of heartwood is linked to variables such as location (elevation, soil fertility, etc.), genetic varieties, and growth characteristics (tree size, diameter at breast height, and growth rate) [16,17,18]. Moreover, the heartwood and sapwood’s radius, area, and volume at breast height are commonly utilized as indicators for comparison and evaluation purposes between trees [7,8,9,19]. Ištok’s analysis of the anatomical attributes of poplar clones revealed considerable differences among distinct locations [20]. The heartwood coloration of *Cryptomeria japonica*, a considerable plantation tree, displays significant heterogeneity among clones [21]. Several chosen triploid *Acacia* hybrid clones (*A. mangium* × *A. auriculiformis*) grow at comparable rates to commercial diploid clones planted in Vietnam [22]. The difference in *Scots pine* tree species can cause growth efficiency variations in the heartwood and sapwood [8,23]. Extensive research has been conducted to determine the growth patterns of sapwood and heartwood in various clones and locations [24,25,26].

The objectives of our study were as follows. First, to increase heartwood yield at stand and clone level by studying the horizontal and vertical distributions and variations. Second, to provide estimated values of genetic gains and clonal repeatabilities for improving and breeding program of blackwood clones. Third, to lay the groundwork for breeding selection of high-grade heartwood of *A. melanoxylon* in South China. To achieve our goal, six 10-year-old *A. melanoxylon* clones, namely, SF1, SR3, SR14, SR17, SR20, and SR25, were thoroughly investigated in different provinces of South China (another two clones, SF1 and SR18, were selected to verify the heartwood volume model). Our findings also provide new insights into understanding heartwood development laws.

## 2. Materials and Methods

### 2.1. Experimental Site Information

Jiuzhou town, BaiSe city, GuangXi Zhuang Autonomous Region (BS) (24°39′ N; 05°46′ W), and ZhongBa town, Heyuan city, Guangdong Province (HY) (23°40′ N; 115°19′ W) were the two sampling sites. Both towns have a subtropical monsoon climate. The average annual temperature of HY is 20.7–22.0 °C and has an annual precipitation of 1768.9 mm. The average annual temperature of BS is 19.0–22.1 °C with an average annual precipitation of 1114.9 mm. These experimental forests are located between 310 and 540 m (310–349 m at HY; 516–540 m at BS) above sea level, with a planting density of 1050 plants per hectare and no cutting (Table 1). In the spring of 2011 in both towns, annual seedlings were selected for afforestation, and a randomized complete block design was adopted, with 4 plots and 3 repetitions, and the plant spacing was 2 m × 2 m. The survey data were collected from the end of December 2021 to the middle of January 2022.

### 2.2. Sampling and Measurements

*A. melanoxylon* plantations in HY and BS Provinces were investigated in December 2021 and January 2022, with at least three plots set for each site factor. In each sample plot, 10 quadrats of 600 m^2^ (20 m × 20 m × 30 m) were established, and the diameter and height of each tree were measured. To record the number of species, individuals, height, and coverage, three 2 m^2^ undergrowth shrub layer plots were placed in the corners and center of each sample plot, and another 1 m^2^ herbaceous plot was placed in the shrub plot.

Then, six clones (SF1, SR3, SR14, SR17, SR20, SR25) of dominant *A. melanoxylon* trees were chosen for stem analysis at two sites (Table 1). Every clone was repeated three times. These six clones are excellent, 10 years old, and available on both sites. Our early study also selected them from more than 1000 trees in this full-sib family [27,28]. Finally, six average trees (SF1, SR18) were chosen to be verified for Paired t Test with heartwood data.

### 2.3. Method of Stem Analysis

**Figure 1:**
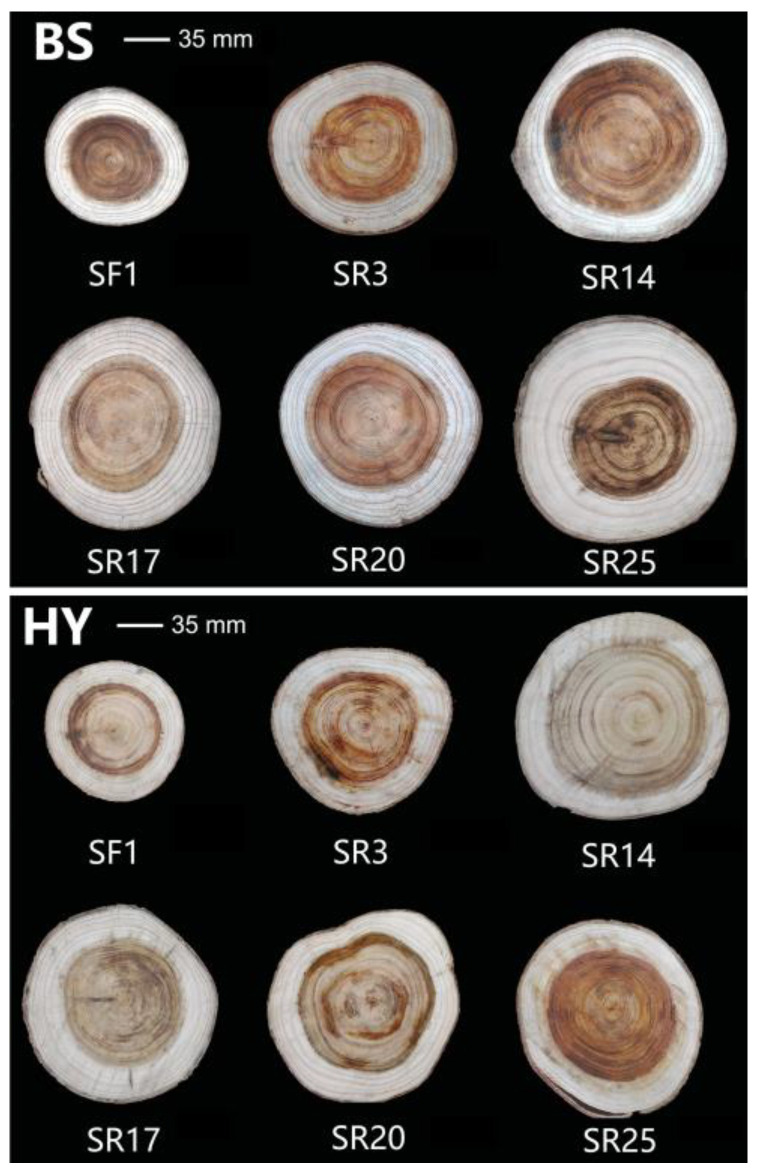
The discs at DBH of six clones at two sites.

**Figure 2:**
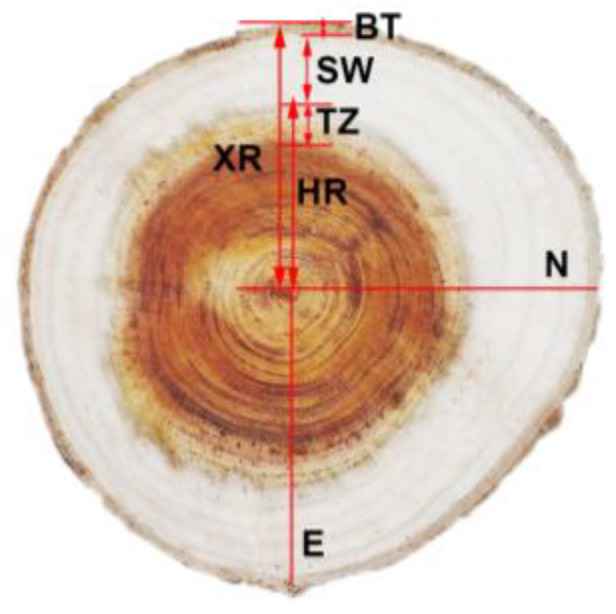
Measurement of heartwood radius and sapwood width of teak discs (XR: xylem radius; HR: heartwood radius; TZ: transition zone; SW: sapwood width; BT: bark thickness; N: north; E: east).

The tree trunk was marked in the east and north directions before being cut down. The average trees were chosen, they were chopped down to the ground diameter and breast diameter, and then a disc with a thickness of 5–7 cm at a length of 2 m was cut until there was less than 1 m remaining. Next, each disc was ground and polished. Discs at the DBH of different clones at two sites are shown in Figure 1. With a firm ruler based on mm, the heartwood radius (HR), sapwood width (SW), and xylem radius were measured in four directions: east, west, north, and south (Figure 2).

### 2.4. Data Analysis

Each disc’s HR, SW, HA (heartwood area), and SA (sapwood area) were calculated as the means of the observation in four directions by scanning with a Wanshen scanner based on cm. A mean sectional area approximate quadrature method estimated individual stem and heartwood volumes. The heartwood volume (HV) and individual volume (V) were calculated based on cm as follows:

(1) $$\mathrm{HV}\;=\sum \left(\frac{1}{2}\times \left(S_{n}+S_{n+1}\right)\times {\;L}_{n}\right)$$

where S_n_ and S_n+1_ are the cross-sectional areas of the base and top heartwood for a stem section, respectively, and L_n_ is the length of the section.

The clonal repeatabilities were calculated within a site as follows:

(2) $$H^{2}=\frac{V_{G}}{V_{P}}=\frac{\sigma_{b}^{2}}{\sigma_{b}^{2}+\frac{\sigma_{e}^{2}}{k}}$$

where *H*^2^ is broad-sense heritability or the clonal repeatabilities which were calculated within a location, *k* is the average value of tree samples per clone within a site, V_G_ is genetic variance, and V_P_ is phenotypic variance [29].

The clonal repeatability of the individual tree *R*^2^ was estimated according to the following equation:

(3) $$R^{2}=\frac{\sigma_{b}^{2}}{\sigma_{b}^{2}+\sigma_{e}^{2}}$$

where $R^{2}$ is the clonal repeatability of the individual tree, *σ_b_*^2^ is the estimated variance of clone, and *σ_e_*^2^ is the variance among the ramets within the clones [30].

The genetic correlation coefficients *r*_A(X,Y)_ between traits at a site were computed using the equation:

(4) $$r_{A\left(X,\;Y\right)}=\frac{\sigma_{b\left(x,y\right)}}{\sqrt{\sigma_{b\left(x\right)}^{2}\sigma_{b\left(y\right)}^{2}}}\;$$

where *σ_b_*_(*x,y*)_ is the clonal component of covariance estimated among traits *x* and *y*, *σ*^2^*_b_*_(*x*)_ is the clonal variance component estimated for trait *x*, and *σ*^2^*_b_*_(*y*)_ is the clonal variance component estimated for trait *y* [24].

These genotype *B* correlations were estimated based on the measured values from different ramets of the exact clone planted in other sites by the following formula:

(5) $$r_{B\left(X,Y\right)}=\frac{r_{p\left(x,y\right)}}{R_{Hx}R_{Hy}}\;\;$$

where *r_p_*_(*x,y*)_ is the coefficient of the phenotypic correlation between the clonal means estimated between *x* measured at site 1 and *y* measured at site 2, and *R_Hx_* and *R_Hy_* are the square roots of the mean repeatability of the clones *x* and *y* estimated at site HY and BS, respectively. *R_H_* is calculated as follows: (*H*^2^ *k*)/(1 + *H*^2^ (*k* − 1)), where *H*^2^ is the individual broad-sense heritability. The SPSS software was used to calculate the phenotypic correlations and the significance [24].

The expected genetic gain in clonal selection was estimated using the equation:

(6) $$\Delta G_{x}=i_{x}\sqrt{R_{x}}\sigma_{P}$$

where *i_x_* is 1.51 (corresponds to the selection of three clones out of 18), *R_x_* is the clonal repeatabilities for trait *x*, and *σ_b_* is the phenotypic standard deviation of clonal means. The expected gain was expressed in percentages of the trait mean at each site [24].

The expected gain in trait *y* was predicted from the correlated response to clonal selection in trait *x* using the following formula:

(7) $$\Delta G_{x}=i_{x}\sqrt{R_{x}}\sigma_{y}r_{xy}$$

where *σ_y_* is the clonal variance component for trait *y*, and *r_xy_* is the genotypic correlation between trait *x* and trait *y* [24]. Correlated genetic response (Δ*G*/*μ* × 100) was expressed in percentages of the trait mean at each site.

Analysis of variance (one-way ANOVA) was used to test the difference in HR, SW, HA, SA, HRP, and HAP at DBH. Graphs were drawn by SigmaPlot 14.0, Adobe Photoshop CC 2019, and Adobe Illustrator CS6.

## 3. Results

### 3.1. The Horizontal Wood Variation within and between Sites

There were significant differences in heartwood radius (HR), heartwood area (HA), heartwood radius percentages (HRP), heartwood area percentage (HAP), and heartwood volume (HV) of *A. melanoxylon* from various sites and clones (*p* < 0.05) (Table 2). As expected, the heartwood data (HR, HA, HAP, and HRP) of SR14 planted in two sites were much more significant than those of the other clones, followed by SR20 and SR17. Meanwhile, the HR, HA, HAP, and HRP of SF1 in BS were higher than those in HY on a horizontal scale. The HR, HA, HAP, and HRP were higher in HY SR14 and SR17 than in BS. The SA, BT, and SW of SR25 in BS were higher than in HY. The SA and SW of SR20 in HY were higher than in BS. In addition, the site had no discernible effect on breast height, bark thickness (BT), sapwood width (SW), or sapwood area (SA). From comparing growth attributes among clones, SR14 is the best clone, and SF1 is the worst. Meanwhile, the SR14, SR25, SR20, and SR17 are much higher than the SF1 and SR3 clones in growth attributes (Table 2).

From the relationship between heartwood and xylem radius at any height along the stem, it was found that the heartwood of *A. melanoxylon* increased rapidly with increasing xylem radius when the xylem radius reached 17–18 mm (Figure S1a). Additionally, HRP quickly increased when the xylem radius expanded from 25 mm to 75 mm. Following that, it gradually increased until it reached a stable value. However, *A. melanoxylon* clones planted in HY were steady at approximately 80%, and clones planted in BS were stable at around 75% (Figure S1b). It is worth noting that regardless of site or clone type, SW had no association with xylem radius (Figure S1c,d). The heartwood area was similarly highly related to the xylem radius at any height along the stem (Figure S2a). The heartwood area percentage grew rapidly to over 50% as the xylem radius expanded from 20 mm to 70 mm and remained nearly stable (Figure S2b). Unlike sapwood width, the sapwood area showed a significant positive association with xylem radius. *A. melanoxylon* clones planted in HY had a flatter trend line than those planted in BS, especially once the xylem width reached 75 mm when the sapwood area of BS increased dramatically with the xylem width (Figure S2c). The sapwood area percentage declined considerably from 20 mm to 80 mm as the xylem radius increased and remained approximately steady at 20–30%. (Figure S2d).

### 3.2. The Vertical Wood Variation within and between Sites

**Figure 3:**
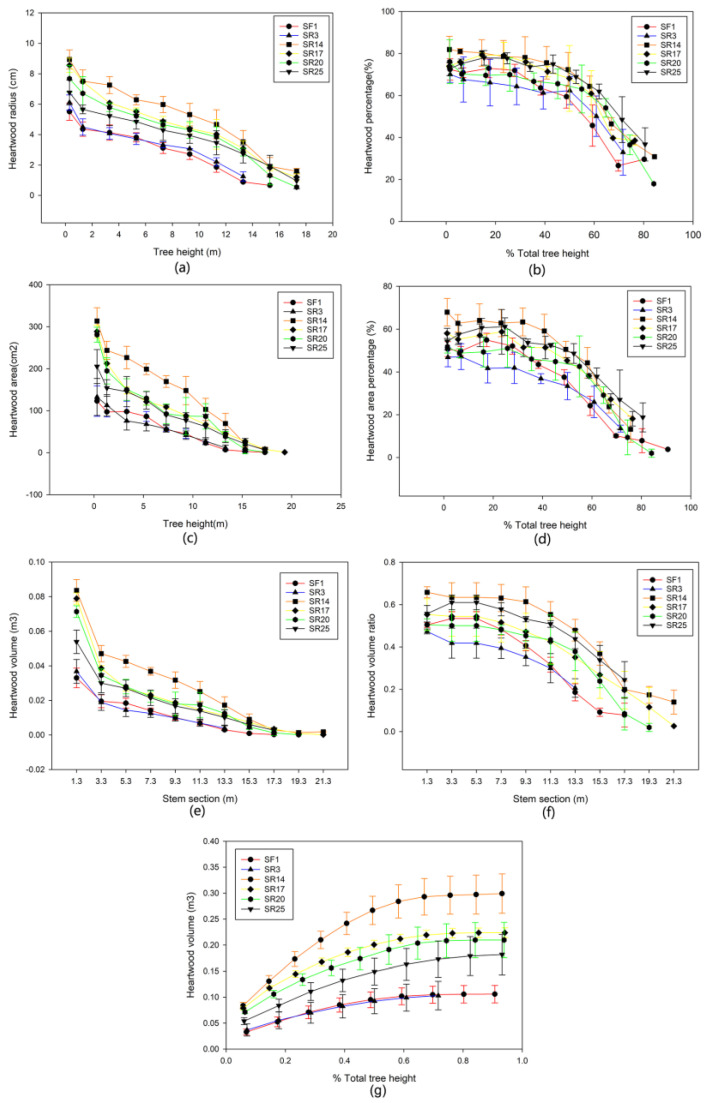
The vertical variations of (**a**) heartwood radius; (**b**) percentage of heartwood radius; (**c**) heartwood area; (**d**) percentage of heartwood area; (**e**) heartwood volume; (**f**) ratio of heartwood volume, and (**g**) cumulative heartwood volume planted in HY with increasing tree height or relative tree height.

The heartwood radius and area decreased with increasing tree height in *A. melanoxylon* planted in HY Province, China, regardless of the clone type (Figure 3a,b). The proportion of heartwood radius in the lower section (<40%) of the stem of SR14, SR17, and SR25 was higher (>73%), and the highest SR14 reached 82% and diminished with increasing tree height, but at a slower rate. The pace of decline was substantially faster in the top region of the stem. Heartwood could reach up to 84.4% of the tree’s height (Figure 3b). The largest heartwood radius and percentage at the same height or relative height of *A. melanoxylon* clones SR14 and SR17 were higher than those of the other clones (Figure 3a,b). Clones had little effect on the heartwood area percentage in the upper part of the *A. melanoxylon* stem. The heartwood area ratio of SR14, SR17, SR20, SR3, and SR25 was substantially higher than that of SF1 at the same relative height of 65% to 75% (Figure 3c,d).

**Figure 4:**
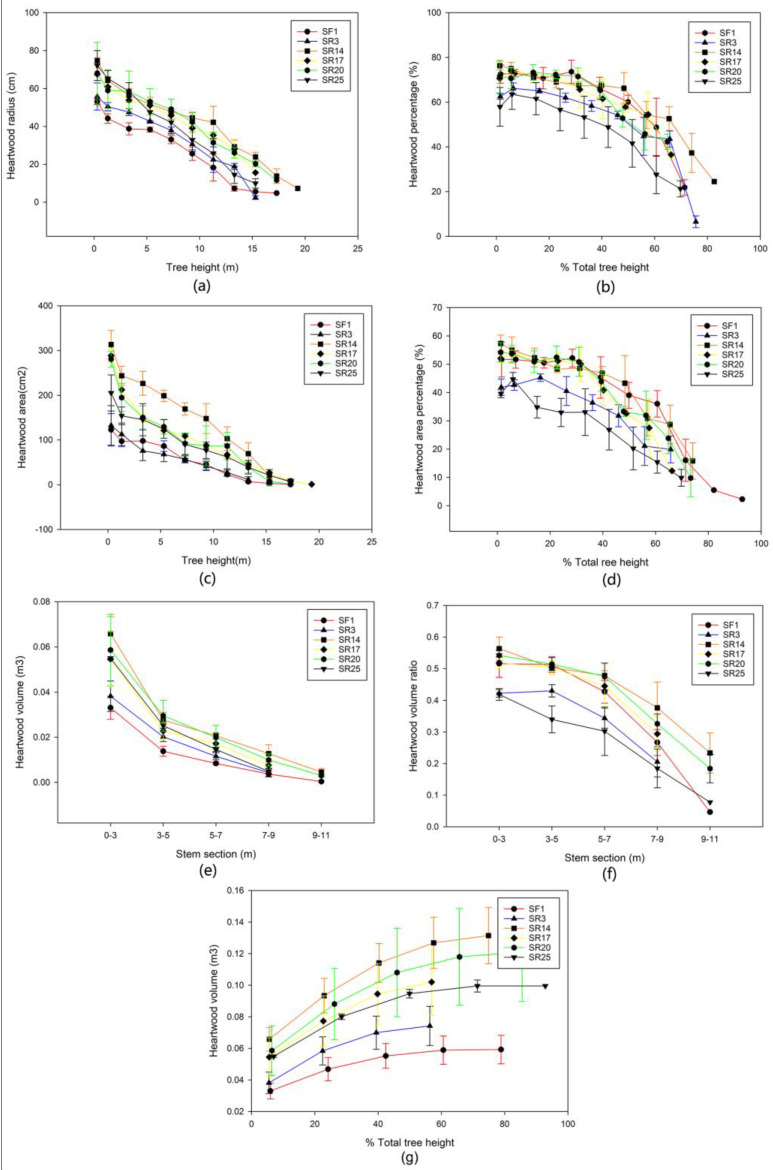
The vertical variations of (**a**) heartwood radius; (**b**) percentage of heartwood radius; (**c**) heartwood area; (**d**) percentage of heartwood area; (**e**) heartwood volume; (**f**) ratio of heartwood volume, and (**g**) cumulative heartwood volume planted in BS with increasing tree height or relative tree height.

Heartwood radius and area decreased with increasing tree height in *A. melanoxylon* planted in BS city, China, regardless of clone type (Figure 4a,b). The proportion of heartwood radius in the lower section of the stem of SR14, SF17, and SR20 was higher (>70%), and it diminished as tree height increased, but at a slower rate. The pace of decline was substantially faster in the top region of the stem. Heartwood may reach a maximum height of 82.6% (Figure 4b). The heartwood radius of *A. melanoxylon* clone SR14 had the most significant proportion of A. melanoxylon clone SR14 at the same or relative height, followed by SR20. It is worth mentioning that SF1 had a more significant percentage of heartwood radius (Figure 4a,b). The heartwood area of several *A. melanoxylon* clones decreased steadily as tree height increased (Figure 4c). The heartwood area ratio and heartwood radius ratio of SR14, SR17, SR20, SR25, and SF1 in the lower section of the stem (<40%) were all higher than those of SR3 at the same relative height (Figure 4b,d). With increasing stem height, heartwood volume decreased significantly (Figure 3e and Figure 4e). Regardless of the site and clone, *A. melanoxylon* was higher in the trunk of 0~33 m, with the highest proportion of SR14 reaching 65.8% (Figure 3e,f and Figure 4e,f). When the tree’s height reached 60%, the increase in heartwood volume came to a stable level, SR14 being the highest, with values of 0.158 in HY and 0.114 in BS (Figure 3g and Figure 4g). The heartwood volumes of SR14, SR17, and SR20 were all higher at the two sites, and the volume ratio of SR25 heartwood in HY was higher than that in BS (Figure 3e and Figure 4e). In addition, in the heartwood volume of these clones, the *A. melanoxylon* growing in HY was slightly larger than that in BS overall.

### 3.3. Relationship between Tree Growth Traits (DBH, DGH, and H) and Wood Attributes

The genotypic and phenotypic correlations are presented in Table 3. The growth characteristics of trees are significantly related to HR, HA, and HV. A significant and positive genetic correlation between HA and HR, SA and SW were observed at each site. Additionally, the phenotypic correlations for SA and HV between the sites were significant and positive, which indicates that the wood properties were more stable than the growth traits. Moreover, a positive estimated genotypic correlation existed between tree growth traits and the wood attributes (BT, SA, HV). These results suggest that selection for growth traits might lead to a slight increase in yield of sapwood and heartwood, since sapwood and heartwood were derived from H, DGH, and DBH. 

The predicted genetic gains from direct clonal selection and correlated genetic responses in heartwood attribution and growth traits are presented in Table 4. The predicted genetic increases were identical in HAP, HRP, and HVP for HY and BS, while the HY site had the highest gain (5.29% in HRP and 9.58% in HAP). Moreover, the HR, HA, and HV increases at sites HY were about twice that of BS, while the gains in SW and SA were about twice that at BS compared to HY. Gains from direct selection in HRP were as high as that of selection for HAP. However, they still resulted in a decrease in SA and BT. Selection for DBH resulted in the same high gains in HR as that of selection for HA and HV. Selection for tree height led to lower HRP, HAP, and HVP increases.

**Figure 5:**
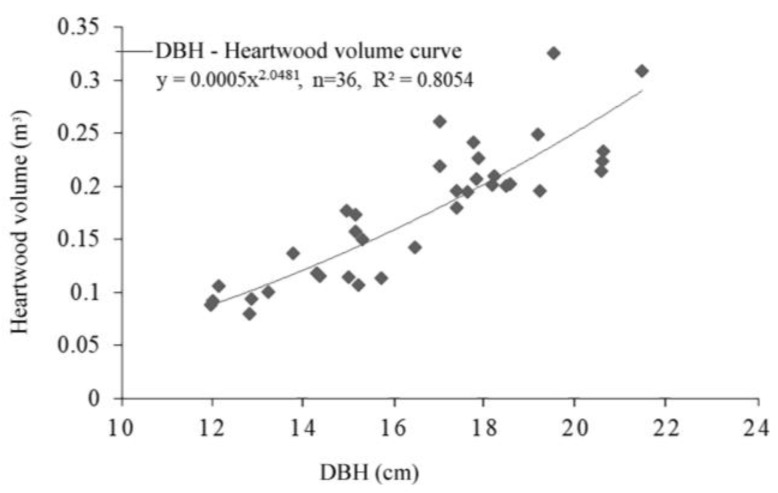
Relationship model between DBH and heartwood volume of *A. melanoxylon*.

DBH is the simplest and most accurate factor in single forest measurements because it is closely related to heartwood and sapwood qualities. As a result, 36 sampling tree ground diameters were chosen to fit a single heartwood volume, and the model was statistically tested. The model (*R*^2^ = 0.8054) can accurately estimate the heartwood volume of *A. melanoxylon* under the verification of six 10-year-old trees (Figure 5, Table 5).

### 3.4. Variation between Growth Traits and Clones

CV is a more appropriate parameter than heritability for comparisons of genetic variation and the ability to respond to selection because it does not depend on the level of residual variation and corrects for different types of scale effects [24]. Our results showed that the most significant coefficient of variation (CV) was H among the eleven indices, reaching 13%. Except for H and DGH, the CV of other traits was 1~7%, and there was no significant variation overall (Table 6). The results suggested no noticeable difference in the economic characteristics of heartwood among clones, which further confirmed the accuracy of our early selection in dominant clones of *A. melanoxylon*. In addition, the differences in growth characteristics among 32 *A. melanoxylon* clones reached a significant level (*p* < 0.05). Broad-sense heritability (*H*^2^) is the proportion of the genetic variance out of the total phenotypic variance in a population. The heritabilities of the eleven indices were between 0.94 and 0.99, and the repeatabilities of the eleven indices were between 0.74 and 0.90. Clonal repeatability of DBH (0.88), H (0.90), HR (0.90), and HVP (0.90) were slightly higher than for SA (0.74), SW (0.75), HAP (0.75), HRP (0.75), HV (0.75), and HV (0.88). The results of repeatabilities between 0.74 and 0.90 in the eleven indices also implied that the growth characteristics of heartwood and sapwood of *A. melanoxylon* clones were less affected by the environment and had substantial heritability.

**Figure 6:**
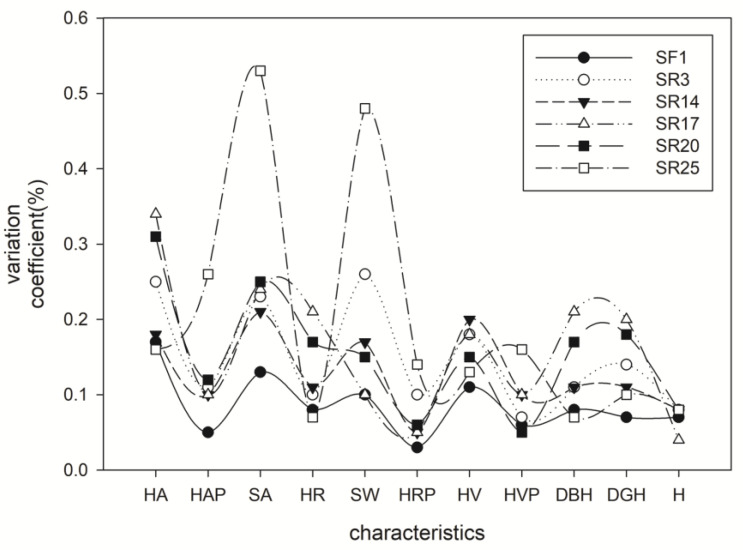
Variation analysis of heartwood characteristics of different clones.

The comparison of variation within clones and between clones is equally important for the law of clonal variation. The statistics in Table 6 and Table 7 show that the variation range between individual plants is more significant than between clones, no matter the tree growth characteristics (DBH, DGH, H), or wood traits of trees. Therefore, the selection range of growth traits in individual plants is more significant than that among clones. Meanwhile, the clone’s SA, SW, and HA variation coefficient is slightly more significant among the 11 growth traits (Figure 6). The variation amplitude within the clone reflects the difference in individual plant growth, and a small amplitude indicates that the increase among individual plants of the clone is neat. Clones SF1, SR3, SR14, SR17, and SR20 have relatively large variation ranges, and SR25 has a relatively large variation range, which may be related to the effect that SR25 is an edge tree species. The marginal tree species were selected because of the shortage of SR25 clones in BS when cutting trees in the experimental forest (Please find all raw data from Table S1).

## 4. Discussion

### 4.1. Horizontal Variation between Clone × Site Interaction

The study on 10-year-old *A. melanoxylon* at two sites showed that clone type significantly affected heartwood radius, heartwood area, heartwood radius percentage, and heartwood area percentage. This study is similar to Bradbury’s research on *A. melanoxylon* [3], but the heartwood and sapwood areas were measured and analyzed for the first time in blackwood. Therefore, compared with a survey on the heartwood of teak, although the heartwood area, sapwood area, and heartwood volume were all significant, the results were similar [9].

When the radius of the xylem is 17–18 mm, heartwood begins to appear, and then there is a significant linear correlation between heartwood radius and xylem radius, while sapwood width has no significant correlation with xylem radius. Xylem radius is a good predictor of heartwood properties [31,32,33]. This study also found that the percentage of heartwood radius gradually increased before the xylem radius reached 70 mm, and the percentage of heartwood radius was basically stable when the stem diameter reached 90 mm. This result is of great significance to improving the management of *A. melanoxylon* plantations and harvesting the most heartwood in a short-term rotation. Perez confirmed that the highest heartwood volume percentage could be obtained by moderately and severely thinning the trunks [34]. 

### 4.2. Vertical Variation between Clone × Site Interaction

The heartwood height of 10-year-old *A. melanoxylon* reached 84.4% of the tree height, close to 85% in the study of *A. melanoxylon* with a 40 cm diameter class planted in Portugal [32]. At the same height or relative height, the heartwood radius and heartwood area of SR14 and SR17 were much higher, followed by those in SR20 and SR25. The heartwood volume was mainly distributed in the middle and lower parts of the trunk (<60% of the tree height), and the cumulative heartwood volume and ratio of SR14, SR17, and SR20 were much higher because the diameter growth of the trees was inhibited in stands with high canopy density. This result was consistent with Tavares’s research on *A. melanoxylon* [32]. The lower part of the trunk is the target area of heartwood production. Combined with the horizontal distribution of heartwood, cutting the lower stem segment with a xylem diameter more significant than 20 cm can maximize the heartwood yield of *A. melanoxylon*.

The vertical fluctuation in the sapwood width of *A. melanoxylon* clones was generally steady at the two sites, primarily between 15 mm and 50 mm. This result was in line with the findings of Tavares on *A. melanoxylon*. The sapwood width remained relatively constant in the middle of the trunk, indicating that wood and trunk radial growth was roughly equal [34]. Although sapwood width varied little throughout the trunk, sapwood area declined dramatically as tree height increased, and the sapwood areas of SR14, SR17, SR20, and SR25 were much more significant than those of the other clones. This result can be similarly interpreted by the fact that the dominant clones had more leaf biomass and area to meet their growth needs [35].

### 4.3. Correlation and Genetic Gains between Sites and Clones

If the correlations between test and planting environments were alike and the objectives of an improvement program were restricted enough to allow selection, then these interactions could be utilized [28]. Generally, an increase in heartwood was related to slightly higher tree growth traits (DBH and H). Previous studies have proved that heartwood increases with increasing tree growth characteristics [9,17]. In our study, the genetic correlations between growth traits and heartwood were positive at two sites, indicating that selection and breeding clone of blackwood not only improves heartwood but also increases tree growth. In this study, no significant phenotypic correlation for heartwood at BS were observed, which suggests that a true G × E interaction exists. The differences between these sites may result in the interaction [28]. However, the phenotypic correlations for SA and HV between the sites were mostly positive, which indicates that the wood properties were more stable than the growth traits.

Site effects represent the response of trees to the combined influences of edaphic and climatic conditions [28]. Even though the current trials were not designed to separate these different effects, some conclusions can still be drawn. Expected genetic gains in this study are based on genotypic correlations, genotypic variances, and clone repeatabilities. In selecting HY using heartwood as the main criteria, high genetic gain in HR (17.24%), HA (30.19%), and HV (28.64%) can be achieved. The poor performance in heartwood at BS may be owing to poor soil and annual precipitation.

For most valuable tree species, heartwood determines the value of the wood [36], but heartwood volume cannot be directly measured in practice. Therefore, accurate and easily measured methods for heartwood volume estimation need to be developed. In this study, the correlation of DBH was the highest among the tree growth factors tested. The heartwood volume model based on DBH fitting could accurately predict the *A. melanoxylon* of different clones. These findings were consistent with Wang’s and Yang’s research and Mossman’s research [9,17,37], where DBH was considered the most effective method for estimating heartwood volume.

### 4.4. Heritability and Repeatability of Clones

Exploring and utilizing the law of genetic variation of forest trees is the premise of genetic improvement, and mastering the law of genetic variation is the basis of formulating breeding strategies [38]. Plant genetic improvement depends on the magnitude of the heritability of economic traits [29,39]. The heritability of *A. melanoxylon* for wood properties was higher than those reported for *A. crassicarpa* and some broadleaved species [25,40,41]. The broad-sense heritability levels of *A. melanoxylon* for tree height and DBH were also higher than those noted for hybrid aspen [42,43]. The high heritability estimates might have resulted from sizeable environmental variation within sites and significant within-tree variation in wood growth. Repeatability can reflect the stability of plant traits, and the more incredible the value, the stronger the stability of features, and the less affected by the environment, the better the selection effect. The repeatability of heartwood traits of blackwood clones generally maintained a high level (>0.74), which indicated that these traits were restricted by strong genetic factors and had high genetic stability, and that the selection based on phenotype was more reliable. This is beneficial to the selection of clones with more heartwood. Strong genetic control of clonal traits is beneficial to maximize the gain, and the repeatability of tree height and DBH is 0.91 and 0.90, respectively, which is consistent with the research that the tree height and DBH of *Pinus tomentosa* clones belong to high repeatability and slightly higher than that of *Pinus radiata* [35]. The difference in genetic control is related to the type and number of research materials, which also shows that it is expected to obtain a better genetic improvement effect by selecting clones with strong genetic control. In a word, the growth and heartwood quality traits of blackwood clones have great genetic improvement potential, which provides the possibility for the evaluation and selection of excellent clones of blackwood.

Our study was based on six genotypes and a relatively small number of testing sites measured over only ten years. Therefore, the results from this study should be further verified by subsequent larger and longer trials. The implication of the G × E interaction for estimating the gain of genetically improved clones of *A. melanoxylon* should be further studied.

## Figures and Tables

**Table 1:** Description of sampled *A. melanoxylon* trees with different clones in two sites.

Sites	Parameters	SF1	SR3	SR14	SR17	SR20	SR25
HY ^1^	Latitude; Longitude	23°40′ N; 15°19′ W					
	Altitude (m)	313–325	310–340	330–336	325–339	347–349	330–347
	Vegetation Situation	*Rhaphiolepis indica*, *Pyracantha fortuneana*, *Carex* spp., *Smilax corbularia*, *Dicranopteris dichotoma*, *Dianella ensifolia*, *Phyllostachys glauca*, *Ardisia japonica*, *Spatholobus suberectus*, *Embelia laeta*, *Melastoma candidum*, *Rourea microphylla*, *Arthraxon hispidus*, *Schima superba.*					
BS ^2^	Latitude; Longitude	24°39′ N;105°46′ W					
	Altitude (m)	531–538	528–539	522–526	522–534	520–522	516–518
	Vegetation Situation	*Maesa japonica*, *Thysanolaena maxima*, *Dicranopteris dichotoma*, *Callicarpa bodinieri*, *Helicteres angustifolia*, *Lygodium japonicum*, *Callicarpa macrophylla*, *Millettia pulchra*, *Cipadessa baccifera*, *Glochidion puberum*, *Ohwia caudata*, *Oplismenus compositus*, *Eupatorium odoratum*, *Tetracera asiatica*, *Parthenocissus tricuspidata.*					

^1^ HY: Zhongba Town, Zijin County, Heyuan City, Guangdong Province, China; ^2^ BS: Jiuzhou Town, Tianlin County, Baise City, GuangXi Zhuang Autonomous Region, China.

**Table 2:** Heartwood, sapwood, and bark thickness attributes of 10-year-old *A. melanoxylon* with different clones planted in HY and BS.

Sites	Parameters	SF1	SR3	SR14	SR17	SR20	SR25
BS	HR ^2^ (cm)	4.42 (0.25) c ^1^	5.05 (0.21) b	6.43 (0.1) a	6.09 (0.59) ab	5.9 (0.11) ab	6.53 (0.41) a
	SW ^3^ (cm)	1.64 (0.15) c	2.58 (0.25) b	2.14 (0.3) b	2.14 (0.39) b	2.40 (0.11) b	3.78 (0.86) a
	BT ^4^ (mm)	2.78 (0.56) c	2.62 (0.25) c	3.10 (0.38) b	3.17 (0.53) b	3.47 (0.52) ab	3.75 (0.20) a
	HA ^5^ (cm^2^)	99.1 (22.83) c	111.90 (9.31) c	183.29 (19.39) a	182.16 (34.63) a	170.8 (21.49) a	174.97 (1.38) a
	SA ^6^ (cm^2^)	91.91 (15.63) c	151.26 (24.45) b	150.47 (23.38) b	156.97 (35.93) b	149.14 (36.31) b	224.01 (9.05) a
	HV ^7^ (m^3^)	0.06 (0.01) c	0.07 (0.01) c	0.13 (0.02) a	0.10 (0.02) b	0.12 (0.03) a	0.10 (0.00) b
	HRP ^8^	0.73 (0.02) ab	0.66 (0.02) c	0.75 (0.03) ab	0.74 (0.02) ab	0.71 (0.05) b	0.64 (0.07) c
	HAP ^9^	0.52 (0.03) ab	0.43 (0.02) b	0.55 (0.04) a	0.54 (0.01) ab	0.54 (0.03) ab	0.44 (0.01) c
	HVP ^10^	0.52 (0.04) b	0.42 (0.01) c	0.56 (0.04) a	0.52 (0.02) b	0.54 (0.00) b	0.42 (0.00) c
HY	HR (cm)	4.36 (0.47) c	4.5 (0.54) c	7.51 (0.74) a	7.48 (0.07) a	6.71 (0.2) a	5.66 (0.28) a
	SW (cm)	1.78 (0.2) c	2.2 (0.87) b	1.77 (0.31) c	2.37 (0.06) b	28.36 (4.62) a	1.88 (0.3) c
	BT (mm)	2.64 (0.53) c	3.28 (0.46) b	3.83 (1.01) a	4.36 (0.36) a	2.71 (0.4) c	3.38 (0.54) b
	HA (cm^2^)	97.02 (12.21) c	112.57 (25.4) bc	243.75 (21.67) a	212.55 (1.55) a	194.93 (31.18) a	154.03 (19.72) b
	SA (cm^2^)	97.84 (9.4) c	125.52 (20.6) b	147.03 (35.41) b	167.35 (20.94) b	209.35 (52.2) a	207.02 a
	HV (m^3^)	0.07 (0.01) c	0.07 (0.01) c	0.18 (0.02) a	0.12 (0.03) a	0.13 (0.02) a	0.11 (0.02) ab
	HRP	0.71 (0.02) b	0.68 (0.10) c	0.81 (0.01) a	0.76 (0.01) a	0.70 (0.04) b	0.75 (0.02) ab
	HAP	0.50 (0.02) ab	0.47 (0.06) c	0.63 (0.04) a	0.55 (0.02) a	0.49 (0.02) c	0.49 (0.02) c
	HVP	0.50 (0.01) b	0.47 (0.01) c	0.66 (0.03) a	0.55 (0.07) ab	0.51 (0.03) b	0.56 (0.04) a

^1^ Numbers in parentheses are the standard error of the mean value; ^2^ HR: heartwood radius at breast height; ^3^ SW: sapwood width at breast height; ^4^ BT: bark thickness; ^5^ HA: heartwood area at breast height; ^6^ SA: sapwood area at breast height; ^7^ HV: heartwood volume; ^8^ HRP: heartwood radius percentage; ^9^ HAP: heartwood area percentage; ^10^ HVP: heartwood volume percentage. Different letters in the same row refer to significant differences between clones in same parameters (*p* < 0.05).

**Table 3:** Phenotypic correlations (below the diagonal, *r_B_*_(_*_x,y_*_)_) and genotypic correlations (above the diagonal, *r_A_*_(_*_X,Y_*_)_) among all traits at individual sites of the six blackwood clones. ^1^ **, *p* < 0.01. ^2^ *, 0.01 < *p* < 0.05. HR, SW, BT, HA, and SA are attributes at breast height.

Sites	Traits	HR	SW	BT	HA	SA	HV	HRP	HAP	HVP	DBH	DGH	H
HY	HR	1	0.65	−0.29	0.99 ** ^1^	0.60	0.20	0.80	0.80	−0.10	0.39	0.35	0.93 **
	SW	0.53	1	−0.09	0.63	0.84 **	0.06	0.54	0.51	−0.42	0.51	0.43	0.44
	BT	0.91 * ^2^	0.32	1	−0.36	0.23	0.55	−0.72	−0.73	0.52	0.57	0.58	−0.19
	HA	−0.91 *	−0.91 *	−0.26	1	0.59	0.22	0.84 *	0.85 *	−0.06	0.37	0.34	0.89 *
	SA	0.79	0.56	0.49	0.62	1	0.59	0.26	0.27	0.14	0.88 *	0.84 *	0.37
	HV	0.65	−0.12	0.44	0.84 *	0.51	1	−0.24	−0.19	0.89 *	0.86 *	0.90 *	0.11
	HRP	−0.38	0.26	−0.78	−0.45	−0.03	−0.43	1	0.99 *	−0.44	−0.11	−0.16	0.68
	HAP	−0.03	0.54	−0.65	−0.12	0.33	−0.21	0.23	1	0.38	−0.09	−0.13	0.67
	HVP	0.15	−0.15	0.09	0.31	0.55	0.78	−0.24	−0.24	1	0.54	0.61	−0.08
	DBH	0.73	−0.09	0.49	0.86 *	0.26	0.55	0.26	0.26	0.84 *	1	0.99 **	0.22
	DGH	0.73	−0.09	0.49	0.86 *	0.26	0.55	0.26	0.26	0.84 *	0.43	1	0.19
	H	0.61	−0.21	0.55	0.80	0.38	0.72	0.09	0.09	0.90 *	0.49	0.61	1
BS	HR	1	−0.27	−0.32	0.95 **	−0.22	−0.44	0.02	−0.30	0.07	−0.34	−0.28	−0.20
	SW	−0.86 *	1	0.62	−0.39	0.97 **	0.42	−0.80	−0.67	−0.72	0.89 *	0.84 *	0.50
	BT	−0.20	0.12	1	−0.57	0.72	0.76	−0.64	−0.38	0.04	0.78	0.81	0.73
	HA	−0.55	0.54	0.84 *	1	−0.36	−0.55	0.12	−0.24	0.05	−0.47	−0.43	−0.33
	SA	−0.85 *	0.06	0.32	−0.80	1	0.53	−0.91 *	−0.76	−0.59	0.93 **	0.90 *	0.60
	HV	−0.15	−0.30	0.44	−0.27	0.15	1	−0.58	−0.40	0.28	0.88 *	0.82 *	0.95 *
	HRP	−0.32	0.65	0.38	−0.39	0.26	0.12	1	0.92 **	0.39	−0.87 *	−0.86 *	−0.66
	HAP	−0.32	0.65	0.38	−0.39	0.26	0.12	0.14	1	0.40	−0.72	−0.72	−0.58
	HVP	0.15	0.03	0.61	−0.09	0.32	0.85	−0.78	−0.65	1	−0.35	−0.26	0.19
	DBH	−0.73	0.03	0.55	−0.80	0.56	0.56	−0.32	0.03	0.45	1	0.99 **	0.83 *
	DGH	−0.50	−0.06	0.44	−0.56	0.44	0.65	−0.49	−0.21	0.51	0.49	1	0.88 *
	H	−0.44	0.48	0.84 *	−0.68	0.79	0.06	−0.14	0.03	−0.36	0.38	0.38	1

**Table 4:** Genetic gains from direct clonal selection (indicated in bold) and correlated genetic response (Δ*G*/*μ* × 100) in wood and tree growth traits of six clones.

Sites	Traits	HR	SW	BT	HA	SA	HV	HRP	HAP	HVP	DBH	DGH	H
HY	HR	**17.24**	10.21	−5.03	17.21	9.26	3.47	13.74	13.87	−1.69	6.69	6.00	16.01
	SW	12.63	**12.67**	−1.26	8.83	10.45	0.84	7.48	7.13	−5.73	7.05	5.95	6.11
	BT	7.82	2.52	**14.02**	−5.06	2.87	7.71	−10.00	−10.23	7.11	7.90	8.04	−2.64
	HA	−8.97	−8.17	−2.62	**30.19**	15.81	6.63	25.06	25.59	−1.76	11.02	10.13	26.61
	SA	26.51	17.01	16.81	20.80	**16.27**	10.79	4.71	4.94	2.50	15.91	15.19	6.72
	HV	11.77	−1.95	7.96	15.11	8.14	**28.64**	−6.81	−5.44	24.87	24.36	25.50	3.13
	HRP	−1.67	1.06	−3.49	−1.95	−0.12	−1.94	**5.29**	5.28	−0.42	−0.58	−0.85	3.60
	HAP	−0.19	3.09	−4.17	−0.76	1.85	−1.33	1.50	**9.58**	7.29	−0.85	−1.23	6.37
	HVP	1.18	−1.08	0.71	2.38	3.82	6.19	−1.88	−1.90	**8.41**	4.61	5.20	−0.68
	DBH	8.53	−0.94	5.84	9.98	2.75	6.52	3.06	3.09	9.71	**13.53**	13.39	2.99
	DGH	7.77	−0.85	5.32	9.10	2.50	5.95	2.79	2.81	8.86	4.65	**12.64**	2.41
	H	2.33	−0.71	2.12	3.01	1.29	2.78	0.33	0.34	3.37	1.87	2.31	**7.47**
BS	HR	**6.30**	−2.00	−2.19	5.07	−1.63	−3.03	0.14	−2.09	0.50	−2.44	−2.00	−1.26
	SW	−17.06	**21.25**	12.20	−5.97	20.66	8.31	−16.43	−13.40	−14.76	18.35	17.25	9.07
	BT	−1.62	1.74	**8.80**	−3.90	6.86	6.73	−5.88	−3.40	0.37	7.19	7.44	5.92
	HA	−3.65	12.21	17.95	**5.70**	−2.85	−4.05	0.92	−1.79	0.38	−3.60	−3.28	−2.23
	SA	−20.50	0.89	4.47	−8.54	**29.13**	14.34	−25.56	−20.79	−16.54	26.22	25.28	14.88
	HV	−2.55	−9.36	12.82	−5.98	4.63	**19.48**	−11.72	−7.88	5.65	17.85	16.57	16.95
	HRP	−1.22	3.44	1.85	−1.44	1.39	0.58	**4.43**	3.97	1.72	−3.87	−3.81	−2.58
	HAP	−1.72	6.02	3.24	−2.52	2.43	1.02	1.31	**6.06**	2.48	−4.49	−4.48	−3.18
	HVP	1.07	0.27	5.23	−0.58	2.96	7.37	−7.02	−5.65	**8.52**	−3.00	−2.22	1.43
	DBH	−7.39	0.40	6.96	−7.69	7.54	7.11	−4.20	0.38	5.89	**11.79**	11.63	8.61
	DGH	−4.73	−0.77	5.31	−5.23	5.74	7.93	−6.27	−2.55	6.44	6.29	**11.06**	8.59
	H	−1.31	3.22	5.33	−3.29	5.36	0.37	−0.96	0.19	−2.36	2.50	2.49	**3.07**

**Table 5:** Paired t Test for observed and predicted data.

Paired Samples	Mean	Standard Deviation	Standard Error	t	Significance Level (*p*)
Observed–Predicted	0.0054	0.01612	0.00806	0.670	0.551

**Table 6:** Variation statistics of growth characteristics among clones.

Character	Min	Max	Range	Mean	Standard Deviation	CV	F Values	Repeatability	Broad-Sense Heritability
HR (cm)	3.94	8.13	4.19	5.75	0.19	0.03	9.10	0.90	0.98
SW (cm)	1.46	4.59	3.13	2.34	0.12	0.05	2.51	0.75	0.94
HA (cm^2^)	66.30	286.30	220.00	155.50	9.32	0.06	7.49	0.88	0.98
HV (m^3^)	0.08	0.33	0.25	0.18	0.01	0.06	21.37	0.75	0.99
HRP	0.55	0.82	0.27	0.71	0.01	0.01	2.82	0.75	0.95
HAP	0.33	0.67	0.34	0.51	0.01	0.03	3.31	0.75	0.95
HVP	0.41	0.69	0.28	0.52	0.01	0.02	7.33	0.90	0.98
DBH (cm)	11.95	22.63	10.68	16.92	0.52	0.03	7.21	0.88	0.96
DGH (cm)	13.3	24.73	11.43	18.86	0.56	0.03	7.04	0.88	0.96
H (m)	17.30	25.50	8.20	16.80	2.12	0.13	2.12	0.90	0.98

**Table 7:** Variation statistics of growth and wood characteristics within clones.

Clones	Character	Range	Mean	Standard Deviation	CV	Clones	Character	Range	Mean	Standard Deviation	CV
SF1	HR (cm)	3.98–4.88	4.39	0.34	0.08	SR3	HR (cm)	3.94–5.28	4.77	0.47	0.10
	SW (cm)	1.53–1.94	1.71	0.17	0.10		SW (cm)	1.58–3.19	2.39	0.61	0.26
	HA (cm^2^)	76.84–122.46	98.06	16.41	0.17		HA (cm^2^)	66.30–122.07	105.57	26.10	0.25
	SA (cm^2^)	73.89–105.81	94.88	11.99	0.13		SA (cm^2^)	83.93–178.30	135.06	30.82	0.23
	HV (m^3^)	0.09–0.12	0.10	0.01	0.11		HV (m^3^)	0.08–0.14	0.11	0.02	0.18
	HRP	0.68–0.75	0.72	0.02	0.03		HRP	0.55–0.74	0.67	0.07	0.10
	HAP	0.47–0.55	0.51	0.03	0.05		HAP	0.38–0.53	0.44	0.05	0.11
	HVP	0.47–0.56	0.51	0.03	0.06		HVP	0.41–0.48	0.45	0.03	0.07
	DBH (cm)	11.95–14.30	12.74	0.92	0.07		DBH (cm)	12.80–16.46	14.92	1.26	0.08
	DGH (cm)	13.30–16.22	14.78	1.11	0.08		DGH (cm)	14.25–18.60	16.26	1.51	0.09
	H (m)	17.80–19.80	12.74	0.92	0.07		H (m)	17.30–20.30	14.92	1.26	0.08
SR14	HR (cm)	6.32–8.13	6.97	0.76	0.11	SR17	HR (cm)	4.49–7.55	6.01	1.23	0.21
	SW (cm)	1.49–2.46	1.95	0.34	0.17		SW (cm)	2.11–2.86	2.49	0.26	0.10
	HA (cm^2^)	164.09–257.39	210.19	38.21	0.18		HA (cm^2^)	93.77–211.00	161.79	55.34	0.34
	SA (cm^2^)	98.28–182.86	146.45	30.94	0.21		SA (cm^2^)	113.65–218.35	160.50	38.88	0.24
	HV (m^3^)	0.20–0.33	0.26	0.05	0.20		HV (m^3^)	0.14–0.23	0.20	0.04	0.18
	HRP	0.72–0.82	0.78	0.04	0.05		HRP	0.66–0.76	0.70	0.04	0.05
	HAP	0.51–0.67	0.59	0.06	0.10		HAP	0.44–0.58	0.49	0.05	0.10
	HVP	0.53–0.65	0.61	0.06	0.10		HVP	0.47–0.60	0.54	0.05	0.10
	DBH (cm)	17.00–19.50	18.54	1.67	0.09		DBH (cm)	13.77–20.57	18.30	3.06	0.17
	DGH (cm)	19.12–23.32	21.01	1.69	0.08		DGH (cm)	15.63–24.73	20.13	3.43	0.17
	H (m)	19.80–25.50	23.12	1.87	0.08		H (m)	21.30–23.50	22.87	0.84	0.04
SR20	HR (cm)	4.75–7.94	1.78	2.60	1.46	SR25	HR (cm)	5.56–6.87	6.07	0.45	0.07
	SW (cm)	2.28–3.12	1.09	1.14	1.05		SW (cm)	1.46–4.59	2.86	1.37	0.48
	HA (cm^2^)	144.55–286.30	43.97	54.89	1.25		HA (cm^2^)	131.39–214.35	167.84	27.49	0.16
	SA (cm^2^)	134.01–230.74	55.37	70.69	1.28		SA (cm^2^)	79.71–351.49	204.98	109.33	0.53
	HV (m^3^)	0.16–0.25	0.10	0.08	0.77		HV (m^3^)	0.15–0.22	0.19	0.03	0.13
	HRP	0.65–0.75	0.28	0.34	1.20		HRP	0.57–0.80	0.69	0.10	0.14
	HAP	0.41–0.57	0.20	0.21	1.05		HAP	0.33–0.56	0.48	0.12	0.26
	HVP	0.49–0.55	0.18	0.23	1.28		HVP	0.41–0.60	0.49	0.08	0.16
	DBH (cm)	15.14–22.63	18.47	2.45	0.13		DBH (cm)	14.95–122.29	18.57	3.24	0.17
	DGH (cm)	15.64–24.21	20.42	2.79	0.14		DGH (cm)	16.35–24.29	20.56	3.15	0.15
	H (m)	19.80–23.80	5.42	8.25	0.08		H (m)	19.80–24.00	21.70	1.70	0.08

## Supplementary Materials

The following are available online at https://www.mdpi.com/2073-4425/14/6/1299/s1, Figure S1: Relationship between xylem radius and (a) heartwood radius, (b) the percentage of heartwood radius, (c) sapwood width, and (d) the percentage of sapwood width; Figure S2: Relationship between xylem radius and (a) heartwood areas, (b) percentage of heartwood areas, (c) sapwood areas, and (d) percentage of sapwood areas. Table S1: The raw data.
