# Correction: Zhang et al. Genotype–Environment Interaction and Horizontal and Vertical Distributions of Heartwood for *Acacia melanoxylon* R.Br. *Genes* 2023, *14*, 1299

**DOI:** 10.3390/genes15020221

**Published:** 2024-02-09

**Authors:** Ruping Zhang, Bingshan Zeng, Tianxiao Chen, Bing Hu

**Affiliations:** Research Institute of Tropical Forestry, Chinese Academy of Forestry, Guangzhou 510520, China; zrpzrpzrp@126.com (R.Z.); b.s.zeng@vip.tom.com (B.Z.); chentianxiao21@126.com (T.C.)

## Table Legend

In the original publication [1], there were several mistakes in the legend for DBH and DGH data shown in Table 1, Table 6 and Table 7. We removed the incorrect data in Table 1 and kept the correct data of the published raw data. We also corrected the data related to DBH and DGH in Table 6 and Table 7. And, the texts related to Table 6 and Table 7 have also been revised. The correct legend appears below.

## Text Correction

There was an error in the original publication. ** indicates that the texts related to Table 6 and Table 7 have also been revised. The corrected texts in the Abstract and Result 3.4 have been revised.

**Abstract:** *Acacia melanoxylon* (blackwood) is a valuable wood with excellent-quality heartwood extensively utilized worldwide. The main aim of this study was to confirm the horizontal and vertical variation and provide estimated values of genetic gains and clonal repeatabilities for improving the breeding program of *A. melanoxylon*. Six blackwood clones at 10 years old were analyzed in Heyuan and Baise cities in China. Stem trunk analysis was conducted for sample trees to explore the differences between heartwood and sapwood. The heartwood radius (HR), heartwood area (HA), and heartwood volume (HV) in heartwood properties decreased as the tree height (H) in growth traits increased, and the HV = 1.2502 DBH (diameter at breast height)^1.7009^ model can accurately estimate the heartwood volume. Furthermore, G × E analysis showed that the heritabilities of the eleven indices, including DBH, DGH (diameter at ground height), H, HR, SW (sapwood width), BT (bark thickness), HA, SA (sapwood area), HV, HRP (heartwood radius percentage), HAP (heartwood area percentage), and HVP (heartwood volume percentage) were between 0.94 and 0.99, and repeatabilities of the eleven indices were between 0.74 and 0.90. Clonal repeatability of DBH (0.88), DGH (0.88), and H (0.90) in growth traits and HR (0.90), HVP (0.90), and HV (0.88) in heartwood properties were slightly higher than for SA (0.74), SW (0.75), HAP (0.75), HRP (0.75), and HVP (0.75). These data also implied that the growth characteristics of heartwood and sapwood of blackwood clones were less affected by the environment and had substantial heritability. 

### 3.4. Variation between Growth Traits and Clones

CV is a more appropriate parameter than heritability for comparisons of genetic variation and the ability to respond to selection because it does not depend on the level of residual variation and corrects for different types of scale effects [24]. Our results showed that the most significant coefficient of variation (CV) was H among the eleven indices, reaching 13%. Except for H and DGH, the CV of other traits was 1~7%, and there was no significant variation overall (Table 6). The results suggested no noticeable difference in the economic characteristics of heartwood among clones, which further confirmed the accuracy of our early selection in dominant clones of *A. melanoxylon*. In addition, the differences in growth characteristics among 32 *A. melanoxylon* clones reached a significant level (*p* < 0.05). Broad-sense heritability (*H*^2^) is the proportion of the genetic variance out of the total phenotypic variance in a population. The heritabilities of the eleven indices were between 0.94 and 0.99, and the repeatabilities of the eleven indices were between 0.74 and 0.90. Clonal repeatability of DBH (0.88), H (0.90), HR (0.90), and HVP (0.90) were slightly higher than for SA (0.74), SW (0.75), HAP (0.75), HRP (0.75), HV (0.75), and HV (0.88). The results of repeatabilities between 0.74 and 0.90 in the eleven indices also implied that the growth characteristics of heartwood and sapwood of *A. melanoxylon* clones were less affected by the environment and had substantial heritability.

The authors state that the scientific conclusions are unaffected. This correction was approved by the Academic Editor. The original publication has also been updated.

## Figures and Tables

**Table 1:** Description of sampled *A. melanoxylon* trees with different clones in two sites.

Sites	Parameters	SF1	SR3	SR14	SR17	SR20	SR25
HY ^1^	Latitude; Longitude	23°40′ N; 15°19′ W					
	Altitude (m)	313–325	310–340	330–336	325–339	347–349	330–347
	Vegetation Situation	*Rhaphiolepis indica*, *Pyracantha fortuneana*, *Carex* spp., *Smilax corbularia*, *Dicranopteris dichotoma*, *Dianella ensifolia*, *Phyllostachys glauca*, *Ardisia japonica*, *Spatholobus suberectus*, *Embelia laeta*, *Melastoma candidum*, *Rourea microphylla*, *Arthraxon hispidus*, *Schima superba.*					
BS ^2^	Latitude; Longitude	24°39′ N; 105°46′ W					
	Altitude (m)	531–538	528–539	522–526	522–534	520–522	516–518
	Vegetation Situation	*Maesa japonica*, *Thysanolaena maxima*, *Dicranopteris dichotoma*, *Callicarpa bodinieri*, *Helicteres angustifolia*, *Lygodium japonicum*, *Callicarpa macrophylla*, *Millettia pulchra*, *Cipadessa baccifera*, *Glochidion puberum*, *Ohwia caudata*, *Oplismenus compositus*, *Eupatorium odoratum*, *Tetracera asiatica*, *Parthenocissus tricuspidata.*					

^1^ HY: Zhongba Town, Zijin County, Heyuan City, Guangdong Province, China; ^2^ BS: Jiuzhou Town, Tianlin County, Baise City, GuangXi Zhuang Autonomous Region, China.

**Table 6:** Variation statistics of growth characteristics among clones.

Character	Min	Max	Range	Mean	Standard Deviation	CV	F Values	Repeatability	Broad-Sense Heritability
HR (cm)	3.94	8.13	4.19	5.75	0.19	0.03	9.10	0.90	0.98
SW (cm)	1.46	4.59	3.13	2.34	0.12	0.05	2.51	0.75	0.94
HA (cm^2^)	66.30	286.30	220.00	155.50	9.32	0.06	7.49	0.88	0.98
HV (m^3^)	0.08	0.33	0.25	0.18	0.01	0.06	21.37	0.75	0.99
HRP	0.55	0.82	0.27	0.71	0.01	0.01	2.82	0.75	0.95
HAP	0.33	0.67	0.34	0.51	0.01	0.03	3.31	0.75	0.95
HVP	0.41	0.69	0.28	0.52	0.01	0.02	7.33	0.90	0.98
DBH (cm)	11.95	22.63	10.68	16.92	0.52	0.03	7.21	0.88	0.96
DGH (cm)	13.3	24.73	11.43	18.86	0.56	0.03	7.04	0.88	0.96
H (m)	17.30	25.50	8.20	16.80	2.12	0.13	2.12	0.90	0.98

**Table 7:** Variation statistics of growth and wood characteristics within clones.

Clones	Character	Range	Mean	Standard Deviation	CV	Clones	Character	Range	Mean	Standard Deviation	CV
SF1	HR (cm)	3.98–4.88	4.39	0.34	0.08	SR3	HR (cm)	3.94–5.28	4.77	0.47	0.10
	SW (cm)	1.53–1.94	1.71	0.17	0.10		SW (cm)	1.58–3.19	2.39	0.61	0.26
	HA (cm^2^)	76.84–122.46	98.06	16.41	0.17		HA (cm^2^)	66.30–122.07	105.57	26.10	0.25
	SA (cm^2^)	73.89–105.81	94.88	11.99	0.13		SA (cm^2^)	83.93–178.30	135.06	30.82	0.23
	HV (m^3^)	0.09–0.12	0.10	0.01	0.11		HV (m^3^)	0.08–0.14	0.11	0.02	0.18
	HRP	0.68–0.75	0.72	0.02	0.03		HRP	0.55–0.74	0.67	0.07	0.10
	HAP	0.47–0.55	0.51	0.03	0.05		HAP	0.38–0.53	0.44	0.05	0.11
	HVP	0.47–0.56	0.51	0.03	0.06		HVP	0.41–0.48	0.45	0.03	0.07
	DBH (cm)	11.95–14.30	12.74	0.92	0.07		DBH (cm)	12.80–16.46	14.92	1.26	0.08
	DGH (cm)	13.30–16.22	14.78	1.11	0.08		DGH (cm)	14.25–18.60	16.26	1.51	0.09
	H (m)	17.80–19.80	12.74	0.92	0.07		H (m)	17.30–20.30	14.92	1.26	0.08
SR14	HR (cm)	6.32–8.13	6.97	0.76	0.11	SR17	HR (cm)	4.49–7.55	6.01	1.23	0.21
	SW (cm)	1.49–2.46	1.95	0.34	0.17		SW (cm)	2.11–2.86	2.49	0.26	0.10
	HA (cm^2^)	164.09–257.39	210.19	38.21	0.18		HA (cm^2^)	93.77–211.00	161.79	55.34	0.34
	SA (cm^2^)	98.28–182.86	146.45	30.94	0.21		SA (cm^2^)	113.65–218.35	160.50	38.88	0.24
	HV (m^3^)	0.20–0.33	0.26	0.05	0.20		HV (m^3^)	0.14–0.23	0.20	0.04	0.18
	HRP	0.72–0.82	0.78	0.04	0.05		HRP	0.66–0.76	0.70	0.04	0.05
	HAP	0.51–0.67	0.59	0.06	0.10		HAP	0.44–0.58	0.49	0.05	0.10
	HVP	0.53–0.65	0.61	0.06	0.10		HVP	0.47–0.60	0.54	0.05	0.10
	DBH (cm)	17.00–19.50	18.54	1.67	0.09		DBH (cm)	13.77–20.57	18.30	3.06	0.17
	DGH (cm)	19.12–23.32	21.01	1.69	0.08		DGH (cm)	15.63–24.73	20.13	3.43	0.17
	H (m)	19.80–25.50	23.12	1.87	0.08		H (m)	21.30–23.50	22.87	0.84	0.04
SR20	HR (cm)	4.75–7.94	1.78	2.60	1.46	SR25	HR (cm)	5.56–6.87	6.07	0.45	0.07
	SW (cm)	2.28–3.12	1.09	1.14	1.05		SW (cm)	1.46–4.59	2.86	1.37	0.48
	HA (cm^2^)	144.55–286.30	43.97	54.89	1.25		HA (cm^2^)	131.39–214.35	167.84	27.49	0.16
	SA (cm^2^)	134.01–230.74	55.37	70.69	1.28		SA (cm^2^)	79.71–351.49	204.98	109.33	0.53
	HV (m^3^)	0.16–0.25	0.10	0.08	0.77		HV (m^3^)	0.15–0.22	0.19	0.03	0.13
	HRP	0.65–0.75	0.28	0.34	1.20		HRP	0.57–0.80	0.69	0.10	0.14
	HAP	0.41–0.57	0.20	0.21	1.05		HAP	0.33–0.56	0.48	0.12	0.26
	HVP	0.49–0.55	0.18	0.23	1.28		HVP	0.41–0.60	0.49	0.08	0.16
	DBH (cm)	15.14–22.63	18.47	2.45	0.13		DBH (cm)	14.95–122.29	18.57	3.24	0.17
	DGH (cm)	15.6424.21	20.42	2.79	0.14		DGH (cm)	16.35–24.29	20.56	3.15	0.15
	H (m)	19.80–23.80	5.42	8.25	0.08		H (m)	19.80–24.00	21.70	1.70	0.08
