# Supplementary figures and images for: Genotype–Environment Interaction and Horizontal and Vertical Distributions of Heartwood for Acacia melanoxylon R.Br
Source: Genes (Basel). 2023 Jun 20;14(6):1299. doi: 10.3390/genes14061299 (PMC10298161; doi:10.3390/genes14061299)

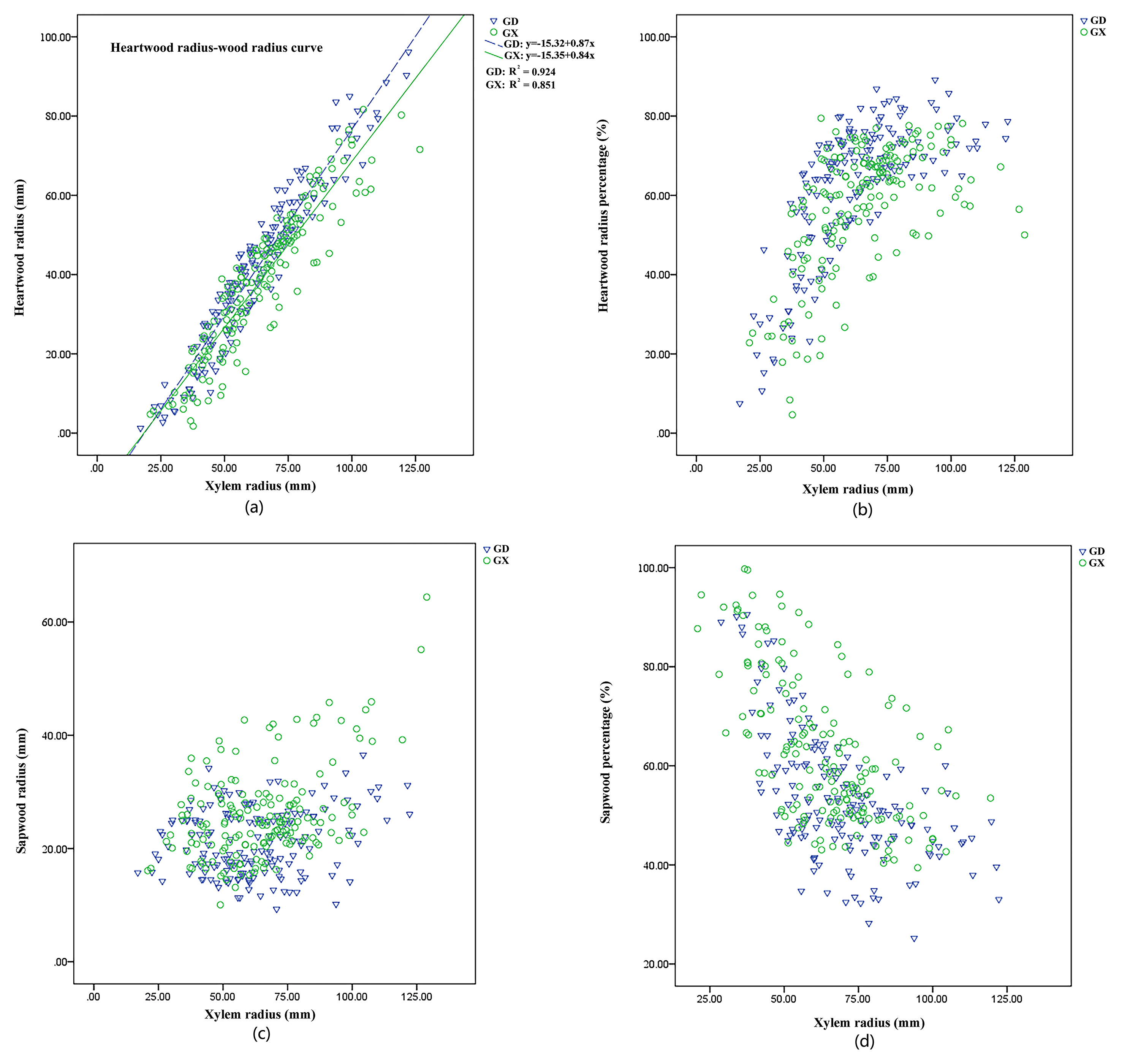

Supplement: Supplementary file 1 [file genes-14-01299-s001.zip › figure S1.tiff]

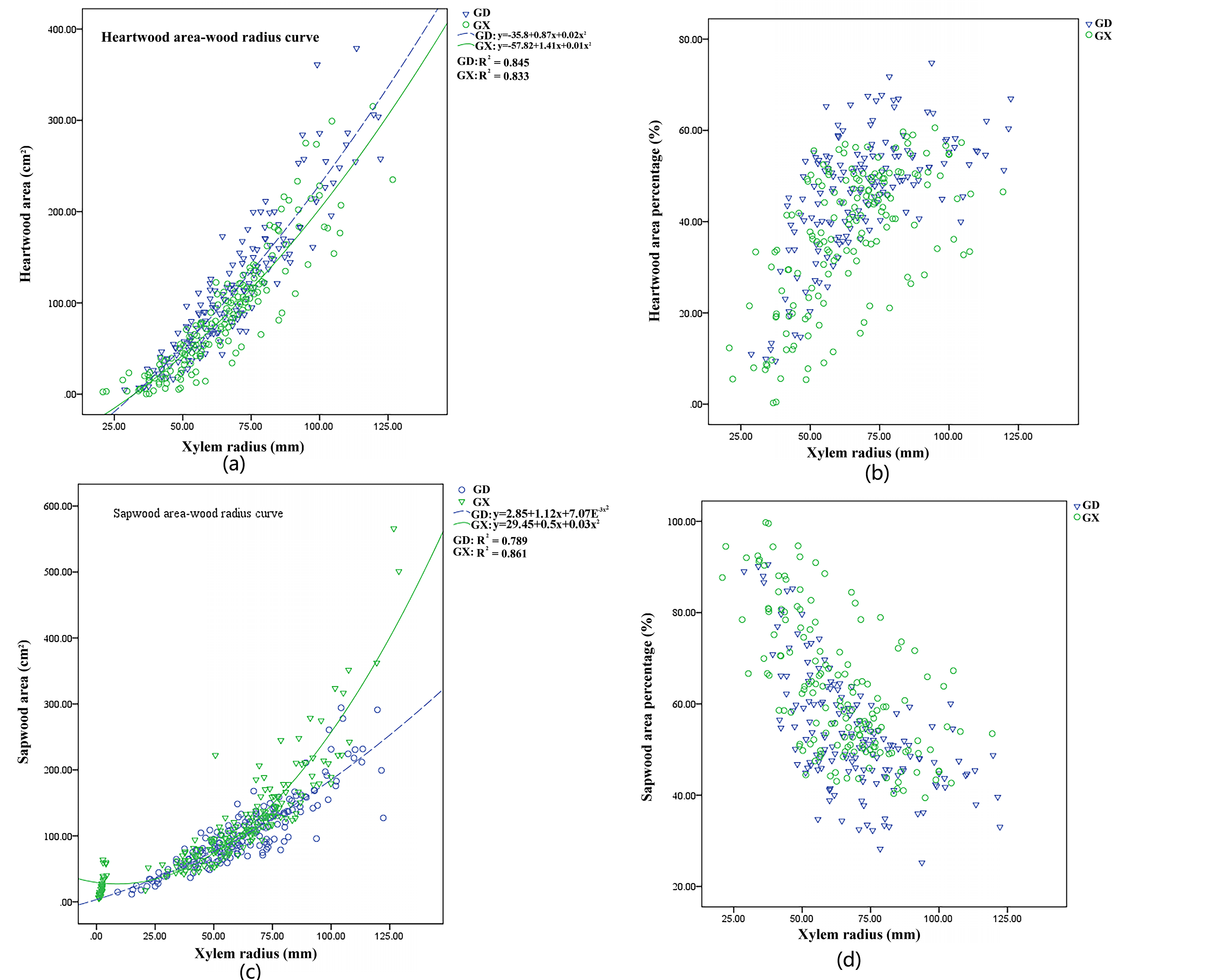

Supplement: Supplementary file 1 [file genes-14-01299-s001.zip › figure S2.tiff]
